# 3, 3′5 Triiodo L Thyronine Induces Apoptosis in Human Breast Cancer MCF-7cells, Repressing SMP30 Expression through Negative Thyroid Response Elements

**DOI:** 10.1371/journal.pone.0020861

**Published:** 2011-06-07

**Authors:** Pranati Sar, Rosalima Peter, Bandita Rath, Alok Das Mohapatra, Sandip K. Mishra

**Affiliations:** 1 Cancer Biology Lab, Department of Gene Function and Regulation, Institute of Life Sciences, Chandrasekharpur, Bhubaneswar, India; 2 Vector Born Disease Lab, Department of Infectious Disease Biology, Institute of Life Sciences, Chandrasekharpur, Bhubaneswar, India; University of Hong Kong, Hong Kong

## Abstract

**Background:**

Thyroid hormones regulate cell proliferation, differentiation as well as apoptosis. However molecular mechanism underlying apoptosis as a result of thyroid hormone signaling is poorly understood. The antiapoptotic role of Senescence Marker Protein-30 (SMP30) has been characterized in response to varieties of stimuli as well as in knock out model. Our earlier data suggest that thyroid hormone 3, 3′5 Triiodo L Thyronine (T_3_), represses SMP30 in rat liver.

**Methodology/Principal Findings:**

In highly metastatic MCF-7, human breast cancer cell line T3 treatment repressed SMP30 expression leading to enhanced apoptosis. Analysis by flow cytometry and other techniques revealed that overexpression and silencing of SMP30 in MCF-7 resulted in decelerated and accelerated apoptosis respectively. In order to identify the cis–acting elements involved in this regulation, we have analyzed hormone responsiveness of transiently transfected h*SMP30* promoter deletion reporter vectors in MCF-7 cells. As opposed to the expected epigenetic outcome, thyroid hormone down regulated h*SMP30* promoter activity despite enhanced recruitment of acetylated H3 on thyroid response elements (TREs). From the stand point of established epigenetic concept we have categorised these two TREs as negative response elements. Our attempt of siRNA mediated silencing of TRβ, reduced the fold of repression of *SMP30* gene expression. In presence of thyroid hormone, Trichostatin- A (TSA), which is a Histone deacetylase (HDAC) inhibitor further inhibited *SMP30* promoter activity. The above findings are in support of categorisation of both the thyroid response element as negative response elements as usually TSA should have reversed the repressions.

**Conclusion:**

This is the first report of novel mechanistic insights into the remarkable downregulation of *SMP30* gene expression by thyroid hormone which in turn induces apoptosis in MCF-7 human breast cancer cells. We believe that our study represents a good ground for future effort to develop new therapeutic approaches to challenge the progression of breast cancer.

## Introduction

The incidence of breast cancer has shown an alarming increase trend in recent years [1]. An estimated 1.7 million women will be diagnosed with breast cancer in 2020 which is a 26% increase from the current levels, mostly in the developing world [2], [3]. The development and growth of many human cancers including breast cancers are known to be influenced by steroid hormones [4], [5]. Abnormal responsiveness of the cells especially to estrogen hormone has been a major cause of breast cancer development and progression [6], [7]. Therefore better understanding and manipulation of the endocrine milieu may provide effective palliative treatment for patients with hormone-dependent cancers [8], [9], [10]. Numerous environmental risk factors, pathological conditions and physiological agents as well as thyroid hormones have been proposed to influence the development of breast cancer [11]. Interestingly, Martinez *et al* reported that the addition of thyroid hormones at non physiological concentrations can alter mammary epithelial cells proliferation [12].

The thyroid gland releases two potent hormones, triiodothyronine (T3) and thyroxine (T4), which can influence and alter the basal metabolism or the oxygen consumption in virtually every cell in the body. However, due to conflicting results regarding the clinical correlation between breast cancer and thyroid diseases, any precise association between thyroid status and the pathogenesis of human breast cancer remains elusive [13]. Similar pathways are shared between thyroid hormone and estrogen in regulating proliferation and growth in the target cells, including cancer cells. So the aberrant signaling by these hormones needs to be evaluated in terms of regulated growth or cancer of the target cells. Receptors of these hormones are critically important in the above process of evaluation. Although the secretion of T4 from thyroid is several times greater than T3, the later is roughly two to three times more effective than the former. T3 binds to specific high affinity receptors called thyroid receptors (TRs) which belong to the super family of nuclear receptors [14] and mediate multiple effects on the phenotype, proliferation and gene expression of cultured normal mammary epithelial cells [15], [16], [17].

The TRs are ligand modulated transcription factors encoded by two genes, TRα and TRβ, located on human chromosomes 17 and 3 respectively [11]. Dependency of human mammary neoplasia on thyroid hormones and controversial reports in literature about the relationship between the thyroid status of the patient and neoplastic illness [18], [19], [20] have suggested that thyroid hormone receptors (TRs) could potentially become a marker and a therapeutic target like the estrogen and progesterone receptors [21]. One of the recent studies reports substantial changes in the expression profile of TRs in breast cancer cells, suggesting a possible deregulation of TRs which could trigger breast cancer development [21], [22], [23]. However, only a few reports have described the presence of TRs in breast tumors [24], [25] and breast cancer cell lines [26], [27]. MCF-7 is one of the well defined breast cancer cell lines where presence of receptors for thyroid hormone has been well documented [26]. This finding makes it a suitable model to study the effect of thyroid hormones in breast cancer.

The effect of thyroid hormone at the cellular level is mediated through TRs, by interacting with thyroid hormone response elements (TREs) in the promoters of the target genes to regulate transcription. Depending on their ability to bind to thyroid hormone, TRs can activate or suppress gene expression in a tissue specific manner through heterodimerisation with retinoid X receptors (RXRs) and interaction with the positive or negative response elements commonly known as TREs in the regulatory regions of target genes [28]. The molecular mechanism of positive transcriptional regulation by TRs is well established. However, the molecular mechanism of negative regulation by nuclear receptors is poorly understood. Several hypotheses have been proposed to explain the action of TR on negative TREs [29]. One hypothesis is that the TR directly regulates transcription through direct binding to target promoter, either to unusual DNA response elements or via protein-protein interactions with other transcription factors associated with cognate response elements. Another hypothesis suggests that the role of TR is indirect mediated through the squelching of coregulators from other transcription factors. Silencing mediator for retinoic and thyroid hormone receptor (SMRT) and nuclear corepressor (NCoR) can increase basal transcription of some target genes in absence of ligand [30]. Coactivator also can play an apparently paradoxical role in T3 dependent negative regulation of some target genes [31]. On the other hand HDACs are recruited by TRs during ligand dependent negative regulation in other cases [32]. Thus, cofactor associated changes in histone acetylation, and alterations in chromatin structure, may be involved in T3 mediated negative regulation. Of note, not all negatively regulated target genes are activated in absence of ligand, suggesting that cofactors may be differentially recruited to promoters of negatively regulated target genes [33]. However there is no consensus over exact mechanism of negative regulation by TRs so far. The response elements may be defined as a negative response element if despite having the basic attribute of an open chromatin conformation such as increased recruitment of acetylated histone recruitment; the outcome is the repression of the corresponding gene.

The role of thyroid hormones in inducing apoptosis in different systems has been well documented in literature [34, 35 and 36]. SMP30 was initially identified as a novel protein that is highly expressed in hepatocytes and in renal tubular epithelia, and its amount decreases with aging [37]. The anti-apoptotic role of SMP30 has been well documented in several reported investigations [38]–[41]. Thus thyroid hormones and SMP30 seem to influence the process of apoptosis in a manner opposite to each other. Hence, it is worth investigating the effect of thyroid hormones on expression and functioning of *SMP30* gene in breast cancer cells in which programmed cell death is deregulated. Additionally, we observed downregulation of SMP30 expression in rat liver by thyroid hormone [42]. Interestingly; MCF-7 breast cancer cells express *SMP30*, which is an antiapoptotic gene.

The mechanism underlying thyroid hormone induced regulation of SMP30 level in breast cancer cells as well as the role of thyroid hormones against breast cancer cells has not been addressed. In our current study, we have attempted to explore the importance of *SMP30* gene, molecular mechanism of its regulation and consequences in response to thyroid hormone treatment in MCF-7 cells. These highly metastatic breast cancer cells are found to have endogenous expression of TRs as well as SMP30. Here, we are providing promising data in support of challenging breast cancer by targeting thyroid hormone receptors which might provide enough supplementary strength to interfere breast cancer metastasis by adjuvant therapies such as Selective estrogen receptor modulators (SERMs).

## Results

### Downregulation of *SMP30* gene in MCF-7 cell line by 3, 3′5 triiodo L thyronine

**Figure 1:**
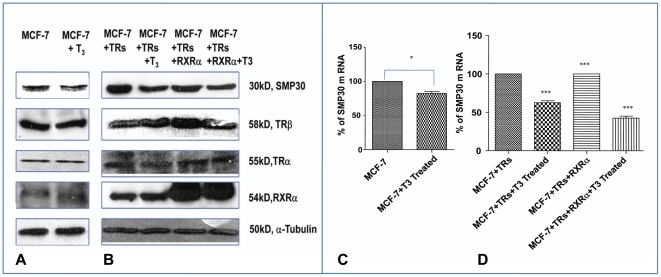
Downregulation of *SMP30* gene in MCF-7 cell line by 3, 3′5 triiodo L thyronine. (A) Representative Western blot of total SMP30 protein in response to overnight treatment with T3 in MCF-7 cells. (B) Expressional analysis of *SMP30* gene after overnight T_3_ treatment to TRs or TRs and RXRα cotransfected MCF-7 cells in Fig.1B was performed by Western blot analysis. 100 µg of protein from whole cell extract was used for Western analysis.In all figures (1A and B) SMP30 protein was detected by SMP30 antibody at ∼30 kDa, TRβ protein was detected by TRβ antibody at 58 kD, TRα protein was detected by TRα antibody at 55 kD and RXRα protein was detected by RXRα antibody at 54 kD were assessed. α-Tubulin was used as a loading control. In Fig. 1C and D, total RNA from cells were harvested and analyzed by quantitative RT-PCR. Shown are the mean values from triplicate samples normalized to GAPDH. CT values obtained from the real time PCR was used to compare the expression label of treated sample from control assuming 100% amplification. Paired student's t test performed; *, *P*<0.01 results were confirmed in three independent experiments as in Fig. 1C. *** *P*<0.0001difference from control using ANOVA as in Fig. 1D.

MCF-7 breast cancer cells express a substantially good amount of SMP30 which is a well established antiapoptotic gene. Here we have examined the effect of thyroid hormone on *SMP30* gene expression both at transcriptional and translational level. Fig.1 shows that overnight treatment of thyroid hormone downregulate SMP30 protein expression in MCF-7 cells. Although TRs are present endogenously in MCF-7 cells, we over expressed them along with RXRα to maintain appropriate amount of functionally active TRs in these breast cancer cells. It is known that thyroid hormone receptors can bind to a TRE as monomers, as homodimers or as heterodimers with the RXR. Although the heterodimer affords the highest binding affinity, and is thought to represent the major functional form of the receptor, it would be interesting to analyze the effect of T3 on SMP30 expression in MCF-7 cells not transfected with RXRα. Expression of SMP30 was significantly downregulated in presence of only TRs but there was no further repression when both TRs and RXRα were overexpressed as shown in Fig.1B. Overexpression of TRs and RXRα in MCF-7 cells were confirmed by Western blot analysis as shown in Fig.1A, B. In addition we also demonstrated that SMP30 expression was significantly repressed at RNA level by T3 treatment in both TRs and RXRα overexpressed MCF-7 cells than MCF-7 mock cells by real time PCR as in (Fig. 1C, D).

### Identification of high affinity TR binding sites within *SMP30* Promoter

**Figure 2:**
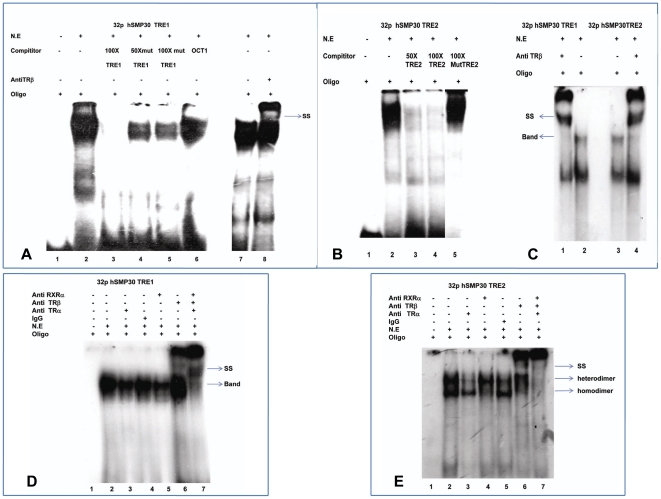
Identification of high affinity TR binding sites within h*SMP30* Promoter. (A) Electrophoretic mobility shift assay for h*SMP30* TRE1 to confirm the binding of TR. lane 1, labeled h*SMP30* TRE1; lane 2, 7 are labeled h*SMP30* TRE1 with 10 µg of Nuclear Extract (N.E.); lane 3–6 describe competition with 100 fold molar excess of self oligo, 50 and 100fold molar excess of mut TRE1 oligo, nonspecific oligo and lane 8 antibody shift with TRβ antibody. (B) Electrophoretic mobility shift assay for h*SMP30* TRE2 to confirm the binding of TR. Lane 1, labeled h*SMP30* TRE2 with 10 µg nuclear extract (N.E); Lane 2–4 describe competition with 50 and 100 fold molar excess of self oligo and mutated TRE2 oligo. (C) As antibody shift was not distinct for short run i.e. for 1 hour, we did long run for two hours, then we got distinct antibody shift in fig. 2C. Lane 2 and 3 are labeled h*SMP30* TRE1 and TRE2 with 10 µg N.E; Lane 1 and 4 are antibody shift with TRβ antibody. (D, E) To confirm the binding of TRβ is specific to h*SMP30* TRE1 and TRE2, we did electrophoretic mobility shift assay in presence of control IgG, TRα, TRβ and RXRα antibodies. Lane 1 labeled h*SMP30* TRE1 and TRE2; lane 2 labeled h*SMP30* TRE1 and TRE2 in presence of 10 µg of N.E of MCF-7 cells - transfected with TRα, TRβ and RXRα expression vectors; lane 3, 4, 5, 6 antibody shift with TRα, normal Rabbit IgG, RXRα and TRβ respectively; lane 7 labeled h*SMP30* TRE1 and TRE2 in presence of 10 µg of N.E incubated with TRα, TRβ and RXRα antibodies in a single reaction. Arrows indicate retarded and supershifted complexes.

**Figure 3:**
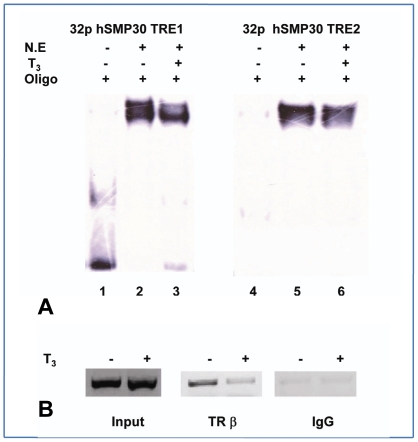
Effects of T_3_ on TR-DNA interaction. (A) Electrophoretic mobility shift assay for h*SMP30* TRE 1 and 2 in presence of T_3_ hormone. Lane 1, 4 are labeled h*SMP30* TRE1 and 2 respectively. Lane 2, 5 are labeled TREs with 10 µg of N.E., Lane 3, 6 are labeled TREs with 10 µg of N.E in presence of 1 µM T_3_ hormone. (B) TRβ binding to *SMP30* promoter is affected by T_3_ treatment was shown in ChIP. After 1 hr. treatment with 1 µM T_3_, MCF-7 cells were crosslinked with 1% formaldehyde and ChIP assays were performed according to manufacturer's protocol (Upstate Biotechnology) with some minor modifications. After reverse crosslinking by heating the samples at 65°C for 4–6 hrs and treating with proteinase K, DNA was elute by phenol chloroform extraction then ethanol precipitation. PCR was performed to visualize the enriched DNA fragments. In vivo association of TRβ protein complex with the h*SMP30* promoter was demonstrated by the amplification of h*SMP30* TREs specific DNA fragments from chromatin complexes precipitated by antibodies for TRβ.

To determine whether there were any TR binding sites within human *SMP30* (h*SMP30*) promoter, we performed an electrophoretic mobility shift assay (EMSA). We have scanned for TREs within 2 kb h*SMP30* promoter from transcription start site. Transcription start site of h*SMP30* gene was analysed by primer extension analysis as shown in Fig. S1. TRs can bind TREs as monomers, homodimers, or heterodimers with the RXR positively or negatively regulate gene expression depending on the nature of TRE and the presence of T3. TREs are generally composed of two or more TR binding half sites arranged as direct repeats spaced by four nucleotides, palindromes without a nucleotide spacer, or inverted palindromes spaced by six nucleotides [43]. The optimal TR binding half site is the octamer TAGGTCA, an extended version of the common core response element hexamer AGGTCA [44]. In h*SMP30* promoter we got two TR binding half sites i.e. at 613 bp and 1.2 kbp from h*SMP30* transcription start site. The sequence of the former from −637 to −600 was found to be ATGTTGGTCAGGCTGGTCTCAAACTCC**TGACCTTA**GG and that of later from −1274 to −1235 was GAAGGACATTAAAGGGACAATTTCTA **TGACCT**GGTG. These two DNA fragments were ^32^P-labeled, incubated with nuclear extract (N.E) of MCF-7 cells - transfected with TRα, TRβ and RXR α expression vectors. The electrophoretic mobility of the radiolabelled DNA fragments was retarded in presence of nuclear extract suggesting possible interaction between the ^32^P-labeled DNA fragments and TRs present in the nuclear extract. This binding was later confirmed by competition with 50 and 100 fold molar excess of cold self oligos, mutated and non specific oligos as shown in Fig.2 A, B. The mobility was further retarded in presence of anti hTRβ antibodies as shown in Fig. 2 A, C, D, E but there was a clear reduction in the intensity of the TR/DNA complex upon addition of anti-TRα and anti RXRα antibodies. When we were adding all the antibodies TRα, TRβ and RXRα together greatly diminished the major protein-DNA complex with a clear shift of band as shown in lane 7 of Fig. 2D and E. Moreover, the binding affinity of TRs to these labelled oligos of two binding sites was also decreased in presence of T3 hormone as shown in Fig. 3A. This result was further confirmed in Chromatin immunoprecipitation (ChIP) assays as in Fig. 3B. The above results clearly indicated that *SMP30* promoter has specific binding sites for TRs.

### *SMP30* Promoter in response to T_3_

**Figure 4:**
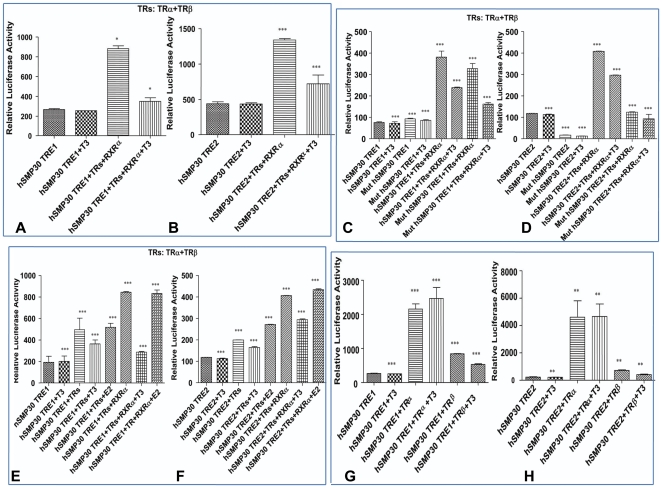
*SMP30* Promoter in response to T_3_. Transient transfections of h*SMP30* TRE1, TRE2 (A, B, E, F, G, H) and mut h*SMP30* TRE1 and TRE2 (C, D) were carried out using MCF-7 cells and similar experiments were also carried out in HEK 293T cell line as shown in Fig.S3. 20 hrs before transfection, cells were plated in DMEM 10%CS media, at a density of 1×10^5^ cells per well in 12 well plates. For transient transfection, 0.5 µg of reporter plasmid DNA, 0. 5 µg of TRβ and TRα (TRs), RXRα expression vector or pCMV vector in (A,B,C,D,E,F) , 0.5 µg of only TRβ or TRα expression vector or pCMV vector in (G, H) ,50 ng of _p_RL-TK control vector were co transfected using Fugene HD transfection reagent (from Roche) as per manufacture's instruction. After 2 4hrs of transfection, cells were subjected to overnight treatment with 1 µM concentrations of T_3_ (A, B, C, D, G, H), 10 nM of E2, 1 µM of T3 or ethanol vehicle to cells (E, F) in 10% CS –DMEM. Then cell lysates were prepared and luciferase activities were measured. Values are the mean of three independent experiments ± SD normalized to Renilla activity. * *P*<0.0232 difference from control using ANOVA for Fig A, *** *P*<0.0001difference from vehicle control using ANOVA in Fig. B, C, D, E, F and G, ** *P*<0.001difference from vehicle control using ANOVA in Fig. H.

We examined the response of T3 on transcriptional activity of h*SMP30* promoter in MCF-7 cells by measuring luciferase activity. We transfected reporter constructs having an oligo representative of the TREs appended to some minimal promoter along with or without expression vectors in MCF-7cells. The minimal promoter (−684 to −455) region cloned in PGL3 basic vector is known as h*SMP30* TRE1 and cloned (−1290 to −1015) promoter fragment is known as h*SMP30* TRE2. Luciferase activity of both the reporter vectors in MCF-7 cells having endogenous TRs did not show any significant difference in presence or absence of T3 where as luciferase activity was induced by over expressing TRs along with RXRα in absence of ligand and repressed in presence of ligand as shown in Fig 4A, B. However there was no repression found in overexpressing only RXRα by T3 treatment as presented in Fig. S2. Similar results were obtained using other cell line such as HEK 293 cells (as Fig.S3). The *SMP30* promoter behaved similarly to those of TSHα [45], SOD1 gene [46], Necdin gene [47] which are negatively regulated by T3. These observations suggested that the TREs on *SMP30* promoter were negatively regulated by T_3_.

The total luciferase expression of mutated construct of h*SMP30* TRE2 (in the presence and absence of T3) was decreased as compared with wild type and the changes in the repression ratios were also small as compared with those noted with the wild type shown in Fig.4C. However, mutated h*SMP30* TRE1 did not show the similar pattern. Repression was still observed although decreased luciferase activity was detected in Fig.4D. These results indicated that high affinity TR binding TREs were not necessary for T3 mediated negative regulation of *SMP30* gene similar to earlier observation in case of some other genes i.e CD44 gene [48], hTSHβ [49], RSV nTRE [50], [51].

To analyze the effect of TRs on h*SMP30* TREs by estradiol (E2) stimulation, we did luciferase assays of h*SMP30* TREs in presence and absence of E2. We found ligand independent or dependent activation of promoter activity in case of both h*SMP30* TRE1 and TRE2.However, in case of thyroid hormone treatment there was downregulation of luciferase activity as shown in Fig. 4E and F.

Luciferase activity of both the reporter vectors h*SMP30* TRE1, TRE2 in MCF-7 cells having endogenous TRs did not show any significant difference in presence or absence of T3 where as luciferase activity was induced by over expressing TRα in absence or presence of ligand. However there was significant repression found while overexpressing only TRβ in presence of ligand as presented in Fig.4G and H. These results indicate that h*SMP30* promoter responds to exogenous expression of TRβ and this effect is synergized by T3 through further repressing the promoter activity.

### Effects of the HDAC inhibitor TSA, on T3 mediated hSMP30 repression

T_3_ is known to recruit HDACs to the thyrotropin releasing hormone (TRH) and thyroid stimulating hormone TSH β promoters during ligand dependent negative regulation. Hence, histone deacetylation may also be an important mechanism for the negative regulation of other target genes by T_3_ [32 and 52].

**Figure 5:**
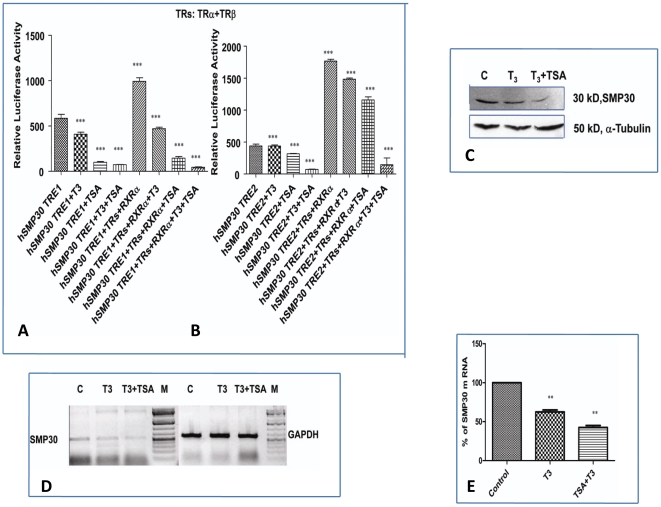
Effects of the HDAC inhibitor TSA, on T3 mediated hSMP30 repression. (A, B)After 24 hrs of transfection as described above, cells were subjected to overnight (1 µM) T_3_ treatment followed by 6 hrs incubation in 330 nM of TSA or ethanol vehicle to control cells. Cell lysates were prepared and luciferase activities were measured. Values are the mean of three independent experiments ± SD normalized to Renilla activity. *** *P*<0.0001difference from vehicle control using ANOVA in Fig. A and B. (C, D, E) Expressional analysis of *SMP30* gene after T_3_ and TSA treatment to MCF-7 cells was analysed by RT-PCR, Western blot analysis and real time PCR. RT-PCR and Real time PCR were preformed as described previously. Western blot analysis was carried as described previously. In Fig. C, SMP30 protein (upper panel) band at ∼30 kDa was detected by SMP30 antibody.α-Tubulin (lower panel) was used as a loading control. A representative RT-PCR data has shown in Fig. D. *SMP30* gene (left panel) and GAPDH gene in (right panel) using cDNA from MCF-7 control cells, T3 treated and T3+TSA treated cells. Same cDNA was used for quantitative RT-PCR as in Fig. E. Shown are the mean of triplicate samples (mean ± SD) normalized to GAPDH. CT values obtained from the real time PCR was used to compare the expression label of treated sample from control assuming 100% amplification. Results were confirmed in three independent experiments. ** *P*<0.0029 difference from control using ANOVA.

We therefore examined the effects of the histone deacetylase inhibitor TSA, on T_3_ mediated SMP30 repression in MCF-7 cells by referring the following papers [32], [47]. The ligand dependent and independent transcriptional activation of the *SMP30* reporter construct was abolished when TSA was added as shown in Fig 5A, B.

Effect of TSA on *SMP30* gene expression in MCF-7 cells was analysed both at RNA and protein level by RT-PCR and Western Blot analysis in Fig. 5C, D and E. T_3_ treatment resulted in repression of *SMP30* gene expression by 40% and TSA further repressed SMP30 expression by 20% as here in Fig. 5E. These results suggested that T_3_ dependent histone acetylation of *SMP30* promoter has an important role in regulation of *SMP30* gene expression. The above results also imply that deacetylation did not affect T3 dependent silencing of transcription of the *SMP30* promoter, but instead was involved in the ligand independent activation, thus reinforcing the differences in the mechanisms behind TR dependent transcriptional regulation of negative thyroid response element (nTREs) vs positive thyroid response element ( pTREs).

### Recruitment of Cofactors to *SMP30* promoter after T_3_ treatment

**Figure 6:**
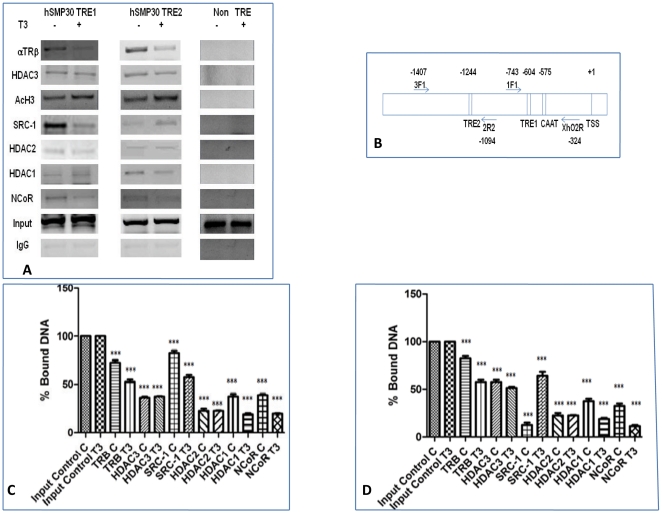
Recruitment of Cofactors to *SMP30* promoter after T_3_ treatment. (A) Corepressor complex and coactivator binding to minimal *SMP30* promoter fragment having TREs and of the control regions having non TREs were analysed by ChIP assays. MCF-7 cells were crosslinked with 1% formaldehyde after 1 hour addition of T3, and ChIP assays were performed according to manufacturer's protocol (Upstate Biotechnology) with some minor modifications. After reverse crosslinking by heating the samples at 65°C for 4–6 hrs and treating with proteinase K, DNA was elute by phenol chloroform extraction then ethanol precipitation. In vivo association of theses protein complexes with the h*SMP30* promoter was demonstrated by the amplification of h*SMP30* TREs specific DNA fragments from chromatin complexes precipitated by antibodies for TRβ, HDAC3, HDAC2, HDAC1, NCoR, anti-acetyl H3 antibody(H3K18) and SRC-1. Specific primers for detection of respective regions (TRE1, −744 to −324; TRE2, −1407 to −1094; non TRE, −344 to +67) were represented. Similar results were obtained in multiple independent experiments. (B) Diagram of human *SMP30* promoter and location of primers in ChIP assays. Two thyroid response elements are located at −604 and −1244 position of h*SMP30* gene from transcription start site (TSS). The upstream primer (1F1) for TRE1 started at −743 bp and the downstream primer (Xho2R) started at −324 from TSS of h*SMP30* gene. The forward primer (3F1) of TRE2 started at −1407 bp and the reverse primer (2R2) started at −1094 bp from TSS of h*SMP30* gene. Primers denoted by arrows and name of each primer are written within a box. CAAT represent the CAAT box. (C, D) Quantitative real-time PCR of ChIP products were carried out by taking same sets of primers as conventional PCR and confirmed that the set of the primers for the real-time PCR yielded a single peak in the 40-cycle procedure. CT values obtained from the real time PCR was used to compare the amplification of bound DNA by different antibodies sample from input control assuming 100% amplification. Values are the mean (mean ± SD) normalized to input levels were compared with those obtained with IgG for at least three independent experiments. ***P<0.0001 difference from control using ANOVA.

**Figure 7:**
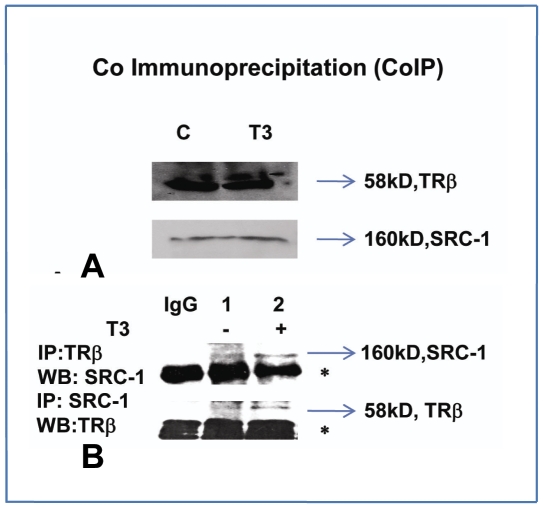
Effects of thyroid hormone on endogenous protein complexes were detected by Co-immunoprecipitation. (A, B) MCF-7 cells were plated in10%CS DMEM, next day cells were subjected to overnight (1 µM) T_3_ treatment. One hundred microgram of whole cell extracts were resolved in SDS-PAGE followed by Western blot analysis as shown in Fig.4E. In Fig.4F, 500 µg of whole cell extracts were precipitated with TRβ, SRC-1 and with normal IgG antibodies. Then precipitated proteins were eluted from protein A/G plus sepharose beads separated by SDS-PAGE, and analyzed by Western blotting (WB). Asterisks denote IgG heavy chain band in upper panel and IgG light chain band in lower panel.

We next investigated the cofactors that participated in the T_3_-dependent histone acetylation of *SMP30* promoter by ChIP analysis (Fig.6). It was found that TRβ can bind to *SMP30* promoter irrespective of the presence or absence of T_3_, but its binding affinity was decreased in the case of former. Here we have measured the binding affinity of thyroid receptor to *SMP30* promoter as a result of changes in the steady state level of the TR bound to DNA across a population of cells. The above finding suggested that interaction of unknown thyroid receptor associated proteins to thyroid receptor bound to negative thyroid response elements were required for the cooperative repression. We examined the recruitment of different cofactors like HDAC3, HDAC2, HDAC1, NCoR and Steroid receptor coactivator (SRC-1) to nTREs by ChIP assays (Fig.6A).Location of primers used in ChIP assays are shown in Fig. 6B. Quantitative real-time PCR of ChIP products were used to compare the amplification of bound DNA by different antibodies sample from input control assuming 100% amplification was shown in Fig.6C and D. There was no further recruitment of HDAC3, HDAC2, NCoR, and HDAC1 on *SMP30* promoter after T_3_ treatment. However, binding of SRC-1 increased with T_3_ treatment on h*SMP30* TRE2 but the same was decreased in h*SMP30* TRE1 in accordance with the reduced binding of TRβ to *SMP30* TREs. But, affinity of SRC-1 to bind TRβ was found to increase with T_3_ treatment as shown in Co Immunoprecipitation (CoIP) Fig. 7A, B. The above observations indicate that histone acetylation of *SMP30* promoter after T3 treatment involves SRC-1 recruitment.

### TRβ enhances TR- mediated Basal Transactivation of the *SMP30* Gene

**Figure 8:**
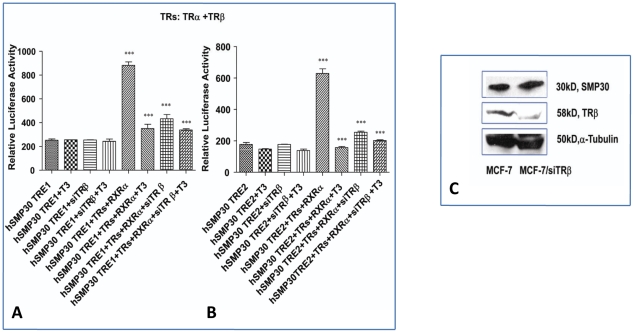
TRβ enhances TR- mediated Basal Transactivation of the *SMP30* gene. (A, B) h*SMP30* TRE1 and TRE2 reporter constructs were transfected in MCF-7 cells. 24 hrs after transfection of reporter plasmid DNA and expression vectors, 200pmole per well of TRβ siRNA or non specific scrambled siRNA (syntesized from Eurogentec) were transfected using oligofectamine according to manufacture's instruction (Invitrogen). After 24 hrs of second round transfection, cells were incubated for an additional 24 hrs in the presence and absence of 1 µM T_3_, then cells were harvested for luciferase assays and readings were taken in duplicates in three independent experiments. Values are the mean of three independent experiments ± SD normalized to Renilla activity. *** *P*<0.0001 difference from control using ANOVA. (C) The endogenous SMP30 expression was measured in TRβ knockdown MCF-7 cells by Western blot analysis. Decrease of TRβ expression by TRβ siRNA transfection was confirmed by Western blot analysis. Western blot analysis was carried by taking equal amount of protein from scrambled siRNA or siTRβ transfected MCF-7cells. SMP30 protein (upper panel) at ∼30 kDa, TRβ protein (middle panel) at ∼58 kDa was detected by TRβ antibody and α-Tubulin (lower panel) was used as a loading control.

Among the two known isoforms of thyroid receptor, TRβ is reported to play a determining role in negative regulation of genes by thyroid hormone [53]. Therefore we examined the role of TRβ in the observed negative regulation of *SMP30* gene by T_3_. Cotransfection of h*SMP30* TRE1 and h*SMP30* TRE2 luciferase construct with TRs and RXRα expression vectors resulted in increased basal transactivation of *SMP30* gene in absence of T_3_. However, in presence of T_3_ the promoter activity of *SMP30* returned to basal level. siRNA mediated silencing of TRβ, completely abolished the basal transactivation of *SMP30* (Fig. 8A, B). T3 dependent repression of SMP30 was released in cells transfected with siTRβ.This result was further confirmed by Western blot analysis. There was (Fig. 8C) slight increase in SMP30 protein expression in the cells transfected with siTRβ compared to scrambled siRNA transfected control cells indicating an important role of TRβ in T3 mediated negative regulation of SMP30.

### Effects of histone acetyl transferase (HAT) inhibitor on *SMP30* promoter transcriptional activity

**Figure 9:**
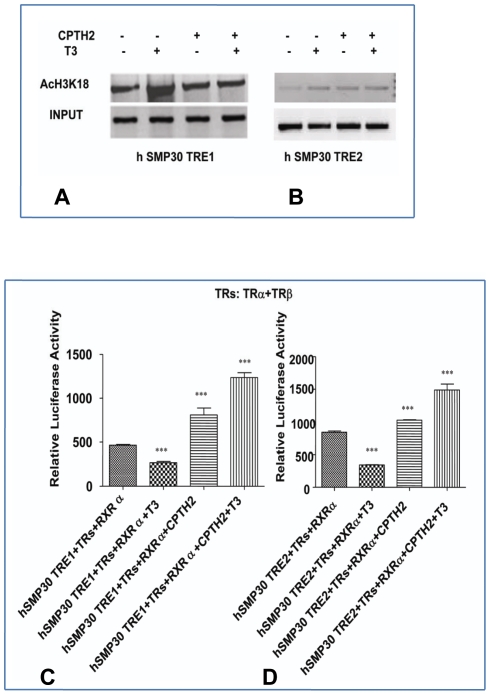
Effects of HAT inhibitors on *SMP30* promoter transcriptional activity. (A) Effects of HAT inhibitor on acetylation of *SMP30* promoter (h*SMP30* TRE1 and h*SMP30* TRE2) was carried out by ChIP assays using anti-acetyl H3 antibody (H3K18). MCF-7 cells were treated with CPTH2 for overnight followed by 1 µM of T_3_ treatment for 1 hr before harvesting for ChIP assays. (B) *SMP30* transcriptional activity after blocking with HAT inhibitor, CPTH2. Transfection of h*SMP30*TRE1 and TRE2 reporter vectors along with expression vectors were carried out as described above followed by overnight treatment with 1 µM of T_3_ and 0.05 mM concentrations of CPTH2 (24 hr post transfection). Then cell lysates were prepared and luciferase activities were measured. Values are the mean of three independent experiments ± SD normalized to Renilla activity. ***P<0.0001 difference from vehicle control using ANOVA.

Our ChIP results using antibodies against histone acetylated lysine at 18position (H3K18) revealed histone acetylation of *SMP30* promoter was increased after T_3_ treatment. T3 causes an increase in acetylation of H3K9 and H3K18 but does not affect acetylation of H3K14 where as H3K27 is deacetylated after T3 treatment [54]. An overall increase in acetylation of H3 leads to negative regulation of TSHα promoter [54]. Our specific finding of distinct recruitment of SRC-1 onTRE2 due to the enhanced association of SRC-1with TRβ in response to T3 signaling as evidenced in Co-IP experiments made out a definitive mechanism behind the repression of *SMP30* gene expression. Distinct withdrawal of SRC-1 from TRE1 supports for the possibility that another cofactors with HAT activity may participate in the negative regulation of *SMP30* gene expression. To further examine the role of coactivator SRC-1 in mediating negative regulation, we examined the effects of HAT inhibitor CPTH-2- [cyclopentylidiene-[4-(4′- chlorophenyl) thiazol-2-yl)hydrazone] on *SMP30* promoter transcriptional activity. CPTH-2, a known modulator of Gcn5 network [55] increased basal transcription levels in presence and absence of T_3_, presumably by decreasing histone acetylation as shown in (Fig.9A, B, C and D). These results provide further evidence for the importance of coactivator SRC-1 recruitment in mediating negative regulation of the *SMP30* gene even though basal levels may be determined by overall histone acetylation.

### Hormonal repression of SMP30 induces apoptosis in MCF-7 cells

**Figure 10:**
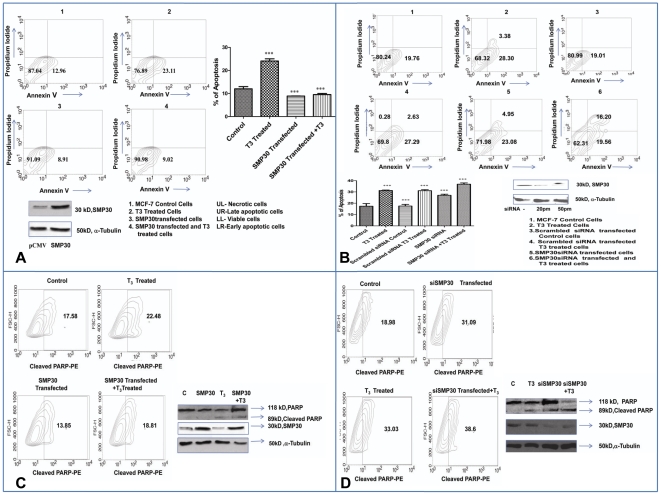
Hormonal repression of SMP30 induces apoptosis in MCF-7 cells. (A, B) Apoptosis was assessed by flow cytometry. MCF-7 cells transfected with mock vector (CMV-FLAG) and vector containing *SMP30* gene as (SMP30-FLAG) were incubated in presence or absence of 10 µM T_3_ for 24 hrs in culture. Knocking down of endogenous SMP30 expression or scambled siRNA transfected control MCF-7 cells were followed by incubation with or without 10 µM T_3_. After 24 hrs of incubation cells were harvested and assayed for apoptosis using the Annexin V FITC apoptosis detection kit, IMGENEX according to manufacturer's instruction. Cells were analyzed in FACS Calibur Analyzer (Becton Dickinson) by using Cell Quest Pro software. Shown are the mean of triplicate samples ± SD normalized to control. Similar findings were found in two other experiments, ***P<0.0001 difference from only vector transfected control using ANOVA in Fig. 6A and***P<0.001difference from scrambled siRNA transfected control using ANOVA in Fig. 6B. (C, D) Detection of apoptosis was further confirmed by flow cytometry analysis of intracellularly stained MCF-7 cells with PE-conjugated polyclonal antibodies against cleaved PARP (BD Biosciences 552933) according to manufacturer's instruction. This was followed by washing with PBS twice and analysis by Flow cytometer. Percentage of population shifting right in the gate represent population of cells showing cleavage of caspase substrate PARP. Cleavage of PARP was demonstrated by Western blot analysis using anti PARP antibody (Calbiochem) taking equal amount of proteins from target cells. Cleavage PARP protein was revealed by presence of two bands, one for full length PARP (119 kD) and another for cleaved PARP (89 kD) in the Western blot. SMP30 protein (upper panel) was detected by SMP30 antibody and the protein band at ∼30 kDa was assessed to determine the efficiency of transfection. α-Tubulin (lower panel) was used as a loading control.

Finally, we investigated whether induction of apoptotic death in MCF-7 cells by thyroid hormone is mediated through down regulation of SMP30 expression. Compared to untreated control, thyroid hormone treatment enhanced the proportion of MCF-7 cells undergoing apoptosis by 50–60% (Fig.10). Overexpression of SMP30 in MCF-7 cells lowered the proportion of apoptotic cells below the basal level (Fig. 10A, C) and knocking down of endogenous SMP30 further enhanced the induction of apoptosis by 20–30% (Fig. 10B, D). Further, Western blot analysis revealed that cleavage of, Poly (ADP Ribose) Polymerase (PARP) an indicator of caspase activation in SMP30 siRNA transfected thyroid hormone treated cells was more in comparison to untreated as well as only thyroid hormone treated cells (Fig. 10D). Similarly, PARP cleavage was found to decrease in SMP30 transfected thyroid hormone treated cells compared to only thyroid hormone treated cells (Fig. 10C). Taken together, the above results clearly suggest that down regulation of SMP30 has an important role during thyroid hormone induced apoptosis in MCF-7 breast cancer cells.

## Discussion

Recent findings indicate that epigenetic alterations of the genome such as acetylation/ deacetylation and other modifications of histones prove to be key factors in breast carcinogenesis [56]. These modifications are quite interesting as targets from therapeutic point of view. Various studies have established that deregulation of hormonal signaling can be correlated to notable epigenetic alterations [57]. The proapoptotic potential of the thyroid hormone has been advocated with evidence by several research findings. [34, 35 and 36]. MCF-7 cells represent one of the few well defined breast cancer cell lines with endogenous expression of TRs [25]. Interestingly, in MCF-7 cells we observed substantially good amount of expression of SMP30 which is well established as an anti-apoptotic gene. The responsiveness of *SMP30* gene to T3 signaling has been reported in an investigation in mouse model [42]. Another recent study demonstrated negative regulation of TSHα gene as a result of T3 signaling [45]. On the basis of the above findings in our current approach, we decided to use MCF-7 as a model cell line of breast cancer to study the effect of thyroid hormones with reference to *SMP30* gene.

In the current study our investigation by flow cytometry revealed that in response to thyroid hormone treatment, apoptosis could be induced in MCF-7 cell line as a result of down regulation of *SMP30* gene expression. In an effort to unravel the underlying mechanism, we performed primer extension analysis to find out the transcription start site of human *SMP30* (h*SMP30*) gene, scanning of h*SMP30* promoter for TRE sites followed by gel retardation assays. On the basis of these experiments we confirmed that there are two important putative TREs at 613 bp as (h*SMP30* TRE1) and 1.2 kbp (h*SMP30* TRE2) from h*SMP30* transcription start site. The luciferase assays which were conducted by taking different deletion constructs of h*SMP30* promoter validated our findings of the putative TRE sites. Looking at the distinct repression caused by liganded TRs (through T3) in Fig. 1, we interpreted that activated TRs play a significant role in downregulating human *SMP30* gene. Similar to our line of study, Guigon *et al* (2011) have identified STAT-5 as a downstream key molecule in the aberrant thyroid hormone signaling emerging out of mutated TRβ. As the consequence of this the mice is predisposed to development of mammary tumors [23]. T47D human breast cancer cell line which is also ER+ve has been used in the above studies. In their investigations remarkable repression of reporter vectors as well as protein were obtained by exogenously transfected TRβ like others similar interesting study. In our current study also significant repression of reporter vectors as well as SMP30 protein were observed by transfecting TRβ. These results indicated that TRα may be important for full activation of nTREs in the absence of T3, possibly by stabilizing interaction with other intermediary factors. But it may be noted with emphasis that it is not important for T3 dependent repression of *SMP30* transcription. The above findings revealed that h*SMP30* promoter responds to exogenous expression of TRβ and this effect was synergized by T3 through further repressing the promoter activity. Silencing of TRβ in MCF-7 cell line reduced the amount of repression as a result of T3 treatment both in terms of protein as well as promoter activity.

The total luciferase expression of mutated construct of h*SMP30* TRE2 (in the presence and absence of T3) was decreased as compared with wild type and the changes in the repression ratios were also small as compared with those noted with the wild type. From this it is quite clear that the decrease in the repression might be due to decrease binding affinity of mutated TREs with TRs. However, mutated h*SMP30* TRE1 did not show the similar pattern. Repression was still observed although decreased luciferase activity was detected. In summary, these results indicated that high affinity TR binding TREs were not necessary for T3 mediated negative regulation of *SMP30* gene. In literature one hypothesis suggests that indirect involvement of TR in T3 mediated negative regulation is through the squelching of coregulators from other transcription factors [29]. The findings were in line with the observations made in the studies of T3 mediated negative regulation of different genes like CD44 [48], hTSHβ [49], RSV nTRE [50], [51]. The results of the experiments, carried out with estrogen signaling, further confirmed our claim that the repression of SMP30 is T3 specific.

The supershift assay performed in presence of TRα, TRβ and RXRα antibodies indicated that TRβ has direct binding affinity for both TRE1 and TRE2. However, it might be possible that TRα, TRβ and RXRα protein forms a heterodimer complex then binds to thyroid response elements of both the sites present in h*SMP30* promoter.

On the basis of our results we interpreted that thyroid response elements cannot be categorically classified as absolute positive or absolute negative elements. However by considering acetylation of histone i.e H3 as bench mark for the above classification we concluded that both TRE1 as well as TRE2 in *SMP30* promoter are negative response element in the context of liganded TRs. By observing both the TREs differentially regulated by TRs we would like to further distinguish them on the basis of different cofactors recruitment on those TREs after T3 treatment. Our specific finding of distinct recruitment of SRC-1 on TRE2 due to the enhanced association of SRC-1with TRβ in response to T3 signaling as evidenced in [46] and also shown in Co-IP experiments made out a definitive mechanism behind the repression of *SMP30* gene expression. Distinct withdrawal of SRC-1 from TRE1 supports the possibility that another coregulator/s with HAT activity may participate in the negative regulation of *SMP30* gene expression. Previous studies in SRC-1 knockout mice and TRβ activation function-2 domain mutant knock in mice suggest that coactivators may be important for negative regulation of genes [58, 59, 60 and 61]. In our investigation another interesting but opposite result was observed in the recruitment pattern of HDAC1, HDAC2 as well as NCoR on TRE1 and TRE2. These differential recruitment patterns also make our above argument more logical. Similar kinds of observations were reported for SOD1, Necdin, and CD44 - target gene that they are under negative regulation by T_3_ [46, 47 and 48]. We believe enhanced recruitment of AcH3 locally does not speak for the complete active chromatin structure. The recent reports by Wang *et al* [45], [54] about TSHα target gene also de-link the traditional concept of enhanced histone acetylation to transcriptional activation of the gene. The above cited investigation also explained our findings of further repression of *SMP30* gene by TSA (Trichostatin A) which is an inhibitor of deacetylases. The repression of *SMP30* gene by liganded TRs was reversed after treatment with HAT inhibitor. From this observation it was very clear that histone acetylation played a major role in the negative regulation of *SMP30* gene by liganded thyroid receptor. It also established the authenticity and importance of our characterisation of two negative thyroid response elements i.e TRE1 and TRE2 of *SMP30* promoter. These observations resemble the functioning of other liganded TR mediated negative TREs (nTREs) [30]. In the above investigation Tagami *et al* discussed that TR mediated recruitment and basal activation by SMRT and NCoR in absence of T3 and reversal of basal activation by dissociation of corepressors in the presence of T3 [30]. On the background of these contrasting results, we advocate that the response of *SMP30* promoter to T3 signaling may not be analysed in isolation with regard to either TRE1 or TRE2. Rather the repression of *SMP30* gene expression is the combined outcome of the response of both the above elements to T3 signaling.

Observations in the present study revealed that thyroid hormone mediated down regulation of SMP30 was direct and accomplished through negative TREs within h*SMP30* promoter. To ascertain the role of SMP30 in the thyroid hormone induced apoptosis of MCF-7 cells, we studied the effect of thyroid hormone after overexpressing and knocking down the *SMP30* gene in MCF-7 cells respectively. Overexpression of SMP30 in our study resulted in reversal of thyroid hormone induced apoptosis of MCF-7 cells while knocking down of SMP30 made the cells increasingly susceptible to thyroid hormone induced apoptosis. These findings indicated anti-apoptotic role of SMP30 in MCF-7 cells which is in accordance with earlier reports regarding the role of SMP30 in literature [38]–[41] in other systems. We found out that apoptosis can be executed by downregulating the expression of *SMP30* gene in MCF-7 cells. Interestingly, this repression was further enhanced as a result of T3 as well as TSA treatment. It is a well established fact that TSA which is a HDAC inhibitor induces apoptosis in different types of cancer [62]. It has also been reported that TSA induced apoptosis in lymphoma cells by decreasing Bcl-2 expression significantly [63]. Overexpression of HDACs increases Bcl-2 expression and inhibition of HDAC activity by HDAC inhibitor decreases its expression [63]. In the specific case we would like to bring several reports about the hypothesis and established finding about the negative thyroid response elements. It has been reported that TSA can inhibit the transcriptional activation of the mouse mammary tumor virus promoter by progesterone, suggesting histone acetylation is not always correlated with increased transcription [64]. Wang *et al* [45] suggested that there is a subset of thyroid hormone mediated negatively regulated genes with increased histone acetylation by microarray analysis. Because of lack of unequivocal evidence correlating TRβ with mammary tumor development a concrete thyroid hormone based therapy has not been developed so far. Similar to our line of observation Guigon *et al* (2011) have shown downregulation of STAT-5 as a result of mutation in TRβ which was manifested in terms of predisposal of the mice to the development of mammary tumors. Our current study and the emerging importance of thyroid hormone in breast cancer support our claim that why we need to seriously think for a combinatorial therapy by taking both T3 as well as other anticancer drugs including those related to TSA [65]. Additionally, downstream target molecules also need to be identified and thoroughly investigated for their role in inducing apoptosis or development of mammary gland tumor as a result of variable or aberrant thyroid hormone signaling. Our current study may prove to be useful for developing potent therapeutic agent to challenge the progression of breast cancer.

## Materials and Methods

### Cell Culture

MCF-7and HEK 293T were obtained from National Centre for Cell Sciences, Pune. They were cultured in DMEM supplemented with 10% fetal bovine serum (FBS) mantained in 5% CO_2_ atmosphere in 37°C until 70–80% confluent. For stimulation with T_3_, culture medium was removed, the cells were rinsed once with phosphate buffer saline (PBS) and medium containing10% charcoal-stripped fetal bovine serum (CS-FBS) was added and incubation continued for 3 days. T_3_ (1 µM), (from Sigma) was diluted in medium and charcoal-stripped 10% fetal bovine serum was added to cells for the times indicated in figure legends.

### Plasmid Constructions

h*SMP30* TRE1 reporter construct was prepared by amplifying human *SMP30* promoter from MCF-7 genomic DNA by using h*SMP* PCR 1F2 and h*SMP* PCR 1R1 primer sequences are shown in table 1. That amplified product, then cloned into _p_Blue TOPO TA vector (Invitrogen). Insert was taken out from _p_Blue TOPO TA vector by HindIII digestion and ligated in to HindIII digested PGL3 Basic vector to obtain h*SMP* TRE1 reporter construct. Similarly, h*SMP* TRE2 reporter construct was prepared by amplifying human *SMP30* promoter from MCF-7 genomic DNA using h*SMP* PCR 2F and h*SMP* PCR 2R1 primer sequences shown in table 1 and then cloned in to _p_TARGET^tm^ vector. Insert was taken out from _p_TARGET^tm^ vector by digesting in Kpn I and Xho I enzyme, ligated in to digested PGL3 basic vector. RT-PCR product of human *SMP30* was prepared from MCF-7 cDNA by using h*SMP30* EcoRI F and XhoI R primers (sequences are shown in table 1). Then that product was ligated in to digested pCMV vector using DNA ligase to make *SMP30* expression vector. _p_CMX-hRXR-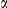, _p_CMX-hTRβ and _p_CMX-rTRα were received from Dr Ronald M Evans, The Salk Institute for Biological Studies, San Diego, CA. All constructs were confirmed by manual sequencing.

**Table 1:** Primers used for cloning.

	Sequence 5′-3′
hSMP PCR 1F2	CACCACGCCCGGCTAATTTTG
hSMP PCR 1R1	GGCAACAAAGTGAGACTTCGTCTC
hSMP PCR 2F	GGCAGTGCCAACATAGAAGG
hSMP PCR 2R1	CTTCCTCAGTGCTGATGTCTCCC
hSMP30 EcoR I F	ACAGAATTCCCTGCGACCATGTCTTCC
hSMP30 Xho I R	ACACTCGAGTCCCGCATAGGAGTAGGGA

### Transfection and Luciferase Assay

Transient transfections were carried out using MCF-7 cells and similar experiments were also carried out in HEK 293T cell lines. 20 hrs before transfection, cells were plated in DMEM 10%CS media, at a density of 1×10^5^ cells per well in 12 well plates. For transient transfection 0.5 µg of reporter plasmid DNA, 0.5 µg of TRβ and TRα as (TRs), RXRα expression vector or pCMV vector and 100 ng of pRL-TK control vector were co transfected using Fugene HD transfection reagent (from Roche) as per manufacture's instruction. After 24 hrs of transfection 1 µM T3 hormone was added and treatment of TSA and HAT inhibitor (from Sigma) were carried out accordingly as shown in figure legend. The cell lysates were prepared, and luciferase activities were measured in duplicates in three independent experiments.

### Transient Transfection of siRNA

24 hrs after transfection of reporter plasmid DNA and expression vectors, 200pmole of TRβ siRNA or nonspecific scrambled siRNA (syntesized from Eurogentec) per well were transfected using oligofectamine according to manufacture's instruction (Invitrogen). After 24 hrs of second round transfection, cells were incubated for an additional 24 hrs in the presence and absence of 1 µM T_3_, then cells were harvested for luciferase assays and readings were taken in duplicates in three independent experiments.

### Electrophoretic mobility shift assays

Electrophoretic mobility shift assay was performed as described in [66]. Oligonucleotides (both strands) corresponding to h*SMP30* TRE sites were synthesized as shown in table 2. For each site one strand was end labelled with γ^32^P ATP using T4 poly nucleotide kinase and annealed to its complementary unlabelled strand. Nuclear extracts of MCF-7transfected with TRα, TRβ and RXRα expression vectors (10 µg) to maintain appropriate concentration of functionally active nuclear receptors were incubated with 20fmoles of radiolabelled oligonucleotide duplex and 1 µg poly (d I-d C) in 30 µl reaction mixture containing 10 mM Tris-HCl (pH 7.5), 50 mM NaCl, 1 mM DTT, 5% glycerol for 20 mins at room temperature. In competition experiments, 100fold molar excess of unlabeled self and mutated oligonucleotide duplexes (sequences as shown in table3) were added during pre incubation period. For antibody shift assay, control IgG (Santacruz), TRα (Abcam), TRβ(Abcam), RXRα (Santacruz) antibodies were added after addition of nuclear extract and probe, followed by incubation for 45 mins at room temperature. Free DNA and protein bound DNA was separated in 5% non-denaturing polyacrylamide gel in 0.5x TBE. After electrophoresis, gels were blotted on to filter paper, dried and autoradiographed.

**Table 2:** Oligonucleotide used for EMSA.

	Sequence 5′–3′
hSMP30 TRE 1 SS	ATGTTGGTCAGGCTGGTCTCAAACTCCTGACCTTAGG
hSMP30 TRE 1 AS	CCTAAGGTCAGGAGTTTGAGACCAGCCTGACCAACAT
hSMP30 TRE 2 SS	GAAGGACATTAAAGGGACAATTTCTATGACCTGGTG
hSMP30 TRE 2 AS	CACCAGGTCATAGAAATTGTCCCTTTAATGTCCTTC

**Table 3:** Mutated Oligonucleotide used for EMSA.

	Sequence 5′–3′
hSMP30 mutTRE1 SS	ATGTTAGTAAGGCTGGTCTCAAACTCCTGAAATTAGG
hSMP30 mutTRE1 AS	CCTAATTTCAGGAGTTTGAGACCAGCCTTACTACAT
hSMP30 mutTRE2 SS	GAAAGAAATTAAAGGGACAATTCTATGAAATGGTG
hSMP30 mutTRE2 AS	CACCATTTCATAGAATTGTCCCTTTAATTTCTTTC

### Chromatin Immunoprecipitation (ChIP) Assay

ChIP assay was performed as previously described [67]. Briefly, MCF-7 cells were grown to 90% confluence in DMEM supplemented with 10% charcoal stripped fetal bovine serum for 3days. After addition of 1 µM T3 for 1 hour, ChIP assays were performed according to manufacturer's protocol (Upstate Biotechnology) with some minor modifications. After reverse crosslinking by heating the samples at 65°C for 4–6 hrs and treating with proteinase K, DNA was elute by phenol chloroform extraction then ethanol precipitation. PCR was performed to visualize the enriched DNA fragments using primers that amplify h*SMP30* TRE1 (h *SMP* PCR 1F1 and Xho2 R), TRE2 promoter (h *SMP* PCR 3F1 and 2R2) and non TRE region. These primer sequences were listed in table 4. Conventional PCR signals stained with ethidium bromide in 2% agarose gel as shown in Fig. 6A.

**Table 4:** Primers used in ChIP PCR.

	Sequence 5′–3′
hSMP PCR 1F1	GGATTCAAGCAATTCTCCTGTCTCAGCC
hSMP XhO2 R	ACACTCGAGACAGTCTGGGCTTTCTCC
hSMP PCR 3F1	CTGCAAGACTCACGGTCTAGCAGGTCATTT
hSMP PCR 2R2	CTTCCTCTACTTCCTCAGTGCTGATGTCTC
hSMP non TRE F	TGGAGAAAGCCCAGACTGTCAGAT
hSMP non TRE R	GGCTGGAAGAATCCTGCAAAG

### Co-immunoprecipitation assays

Co-Immunoprecipitation (Co-IP) and western blot assays were performed as described [68]. Five hundered micrograms of MCF-7 whole cell extracts were subjected to immunoprecipitation (IP) with the indicated antibodies as shown in Fig.7B and the precipitated protein complexes were subjected to Western blot analysis.

### Real-time PCR

MCF-7 cells were harvested after 15 hrs of T3 followed by 6 hrs of TSA with or without treatment. RNA was isolated and *SMP30* and GAPDH mRNA expression were determined by quantitative RT PCR using SYBR greensystem (SIGMA). Relative values (mean ± SD) normalized to GAPDH expression.

### RT PCR

RT-PCR was preformed as described elsewhere [42]. The sequences of sense and antisense primer for h*SMP30* were given in table 5. As a positive control, a fragment of human GAPDH cDNA was amplified by using primers were given in table 5. The PCR condition for *SMP30* was as follow: initial denaturation (94°C 3 min), (94°C 45 s, 65°C 45 s, 72°C 45 s) for 30 cycles and additional extension was per`formed at 72°C for 5 min. The PCR condition for GAPDH as follows: initial denaturation (94°C 3 min), (94°C 45 s, 56°C 45 s, 72°C 45 s) for 30 cycles and additional extension was performed at 72°C for 5 min. Then PCR products were electrophoresed in 1.5% agarose gel.

**Table 5:** Primers used for RT- PCR.

	Sequence 5′–3′
hSMP30 +560 to +580	GCCACCATTGGAACCAAGTT
hSMP30 +1105 to +1085	CCCTCCAAAGCAGCATGAAG
hGAPDH SS2	GATCATCAGCAATGCCTCCT
hGAPDH AS2	TTCCTCTTGTGCTCTTGCTG

### Western Blot Analysis

Whole cell extracts were prepared from cells and Western blotting was performed as described elsewhere [42]. 100 µg of protein from whole cell extract was used for Western analysis. Goat SMP30 antibody (Santacruz) 1:500 times, cleaved PARP (Calbiochem) 1∶1000 times, TRβ (Abcam) 1∶1000 times, SRC-1 (Milipore) 1∶2000 times, TRα (Abcam) 1∶1000 times, RXRα (Santacruz) 1∶1000 times dilution and their respective HRP conjugated secondary antibody (Santacruz) 1∶5000times dilution were used to detect protein expression in western blot. α- Tubulin antibody (Santa Cruz) 1∶1000times dilution was used for immunodetection of α- Tubulin protein, which was used as a loading control. Western Blotting Luminol Reagent kit (Amersham) was used for visualization of bands according to manufacturer's protocol.

### Flow cytometric analysis

To detect intact, necrotic and apoptotic cells, flow cytometry was performed. MCF-7 cells transfected with mock vector (CMV-FLAG) and vector containing *SMP30* gene as (SMP30-FLAG) were incubated in presence or absence of 10 µM T_3_ for 24 hrs in culture. Knocking down of endogenous SMP30 expression or non specific scrambled siRNA was followed by incubation of MCF-7 cells with or without 10 µM T_3._ After 24 hrs of incubation cells were harvested and assayed for apoptosis using the Annexin V FITC apoptosis detection kit, IMGENEX according to manufacturer's instruction. Cells were analyzed in FACS Calibur Analyzer (Becton Dickinson) by using Cell Quest Pro software.

Detection of apoptosis by Annexin-V-FITC detection kit was further confirmed by intracellular staining with PE-conjugated polyclonal antibody against cleaved PARP (BD Biosciences 552933) according to manufacturer's instruction. This was followed by washing with PBS twice and analysis by Flow cytometer. Percentage of population shifting right in the gate represent population of cells showing cleavage of caspase substrate PARP.

### Site-directed Mutagenesis

Two bases of the TREs were mutated as shown in the table 6 (bases in bold letter represents mutated base). For mutagenesis of TREs in hSMP30TRE1 and TRE2 reporter vectors two sets of PCR were carried out using the following combination of primers: ForTRE1: hSMP PCR1F2/ hSMP mut TRE1 AS antisense and hSMP mut TRE1 SS/ hSMP PCR1R1 antisense; for TRE2: hSMP mut TRE2 sense/ hSMP PCR2R1antisense and hSMP PCR2F1 sense/hSMP mut TRE2 antisense; The PCR amplification for mut TRE1was performed using step cycles (94°C for 30 s, 65°C for 30 s, 74°C 30 s) for 35 cycles with a final extension at 74°C for 10 minutes. The PCR amplification for mut TRE2 was performed using step cycles (94°C for 30 s, 60°C for 30 s, 72°C 30 s) for 35 cycles with a final extension at 72°C for 10 minutes. Both the PCR products were purified using QIAquick Gel Extraction Kit. DNA was eluted using 30 µl of autoclaved deionised water. 30 ng of each PCR product was used as a template for the second round of PCR. For example: for construction of mutant TRE1site: 30 ng each of the PCR product hSMP PCR1F2/ hSMP mut TRE1 AS antisense and hSMP mut TRE1 SS/ hSMP PCR1R1 antisense. Similarly mutant TRE2 was made. PCR amplification was carried out using the same parameters as mentioned above. Then the PCR products were purified using QIAquick Gel Extraction Kit. The fragments with mutated TRE sites, having KpnI and XhoI restriction sites and pGL3-Basic vector were digested with KpnI and XhoI enzyme. The fragments were then ligated into restriction enzyme digested pGL3-Basic vector using DNA ligase. The cloned fragments were then confirmed by vector specific PCR using RV3 and GL2 primer, and the mutation was confirmed by sequencing.

**Table 6:** Primers Used for site directed mutagenesis.

	Sequence 5′–3′
hSMP PCR 1F2	CACCACGCCCGGCTAATTTTG
hSMP30 mutTRE1 SS	ATGTTAGTAAGGCTGGTCTCAAACTCCTGAAATTAGG
hSMP30 mutTRE1 AS	CCTAATTTCAGGAGTTTGAGACCAGCCTTACTACAT
hSMP PCR 1R1	GGCAACAAAGTGAGACTTCGTCTC
hSMP PCR 2F	GGCAGTGCCAACATAGAAGG
hSMP30 mutTRE2 SS	GAAAGAAATTAAAGGGACAATTCTATGAAATGGTG
hSMP30 mutTRE2 AS	CACCATTTCATAGAATTGTCCCTTTAATTTCTTTC
hSMP PCR 2R1	CTTCCTCAGTGCTGATGTCTCCC

## Supporting Information

Figure S1 **Determination of h** ***SMP30*** **Transcription start site (TSS).** Determination of transcription initiation sites by primer extension analysis. A Lane 1–4, sequencing reactions; lane 5, primer extension product of MCF-7 total RNA, lane 6, labeled DNA marker from Promega. The sequence corresponding to the transcription start site has been marked by a line and is complementary to the sequencing reactions shown in the figure. B. A schematic diagram of human *SMP30* promoter showing two TREs, CAAT box and transcription start site.
(DOC)

Figure S2 **To analyze the effect of T3 on** ***SMP30*** **Promoter activity in MCF-7 cell in relation to RXRα.** Transient transfections of h*SMP30* TRE1, TRE2 were carried out using MCF-7 cells. 20 hrs before transfection, cells were plated in DMEM 10%CS media, at a density of 1×10^5^ cells per well in 12 well plates. For transient transfection, 0.5 µg of reporter plasmid DNA, 0.5 µg of TRβ and TRα (TRs), RXRAαexpression vector, 100 ng of _p_RL-TK control vector and only vector to control cells were co transfected using Fugene HD transfection reagent (from Roche) as per manufacture's instruction. After 24 hrs of transfection, cells were subjected to overnight treatment with 1 µM T3 and vehicle to control cells in 10% CS –DMEM. Then cell lysates were prepared and luciferase activities were measured. Values are the mean of three independent experiments ± SD normalized to Renilla activity. *** *P*<0.0001difference from vehicle control using ANOVA.
(DOC)

Figure S3 **Luciferase activity of** ***SMP30*** **TREs in HEK293 cell line.** Transient transfections of h*SMP30* TRE1, TRE2 were carried out using HEK 293 cells. 20 hrs before transfection, cells were plated in DMEM 10%CS media, at a density of 1×10^5^ cells per well in 12 well plates. For transient transfection, 0.5 µg of reporter plasmid DNA, 0.5 µg of TRβ and TRα (TRs), RXRα expression vector, 100 ng of _p_RL-TK control vector and only vector to control cells were co transfected using Fugene HD transfection reagent (from Roche) as per manufacture's instruction. After 24 hrs of transfection, cells were subjected to overnight treatment with 1 µM T3 and vehicle to control cells in 10% CS –DMEM. Then cell lysates were prepared and luciferase activities were measured. Values are the mean of three independent experiments ± SD normalized to Renilla activity. ***P<0.0001difference from vehicle control using ANOVA.
(DOC)
