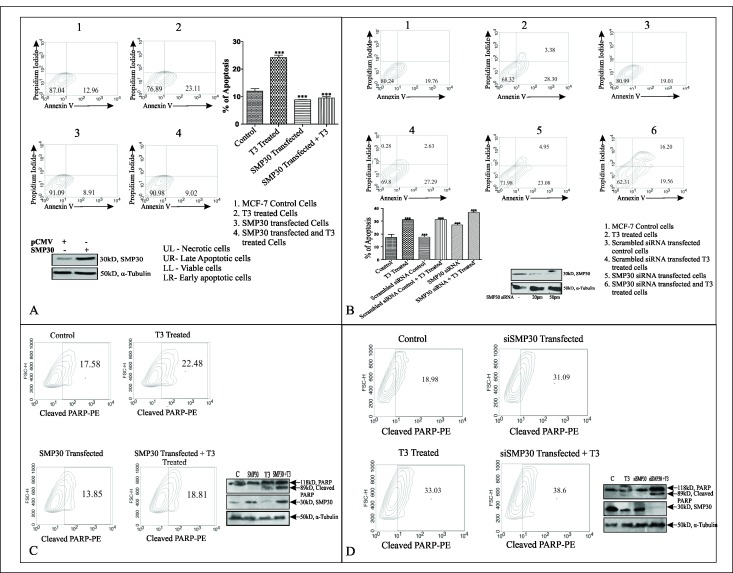# Correction: 3, 3′5 Triiodo L Thyronine Induces Apoptosis in Human Breast Cancer MCF-7cells, Repressing SMP30 Expression through Negative Thyroid Response Elements

**DOI:** 10.1371/annotation/7645d066-aa98-45d6-8c3e-3a30d9e03e4d

**Published:** 2013-06-03

**Authors:** Pranati Sar, Rosalima Peter, Bandita Rath, Alok Das Mohapatra, Sandip K. Mishra

Figure 3B was intentionally re-displayed in Figure 6A (top panel) with the aim of facilitating the interpretation of the data related to the recruitment of different cofactors against the background of recruitment of TR beta. This duplication should have been noted in the article.

The Western Blot data in Figure 10C and 10D were generated by performing a combined experiment for different samples. We acknowledge that PARP, SMP30 as well as alpha tubulin bands were duplicated in a few lanes in both panels as there were common control and T3 treated samples. Upon re-evaluation of the data, we identified several errors in the preparation of these figures and as a result, we have repeated the experiments for these figures and we are providing a revised Figure 10. The data reported in the revised figures do not affect the results described in the published article. In the new experiments we have used anti PARP antibody from Santacruz Biotech, USA.

Revised Figure 10: 

**Figure:**