# Supplementary material for: 3, 3′5 Triiodo L Thyronine Induces Apoptosis in Human Breast Cancer MCF-7cells, Repressing SMP30 Expression through Negative Thyroid Response Elements
Source: PLoS One. 2011 Jun 7;6(6):e20861. doi: 10.1371/journal.pone.0020861 (PMC3110202; doi:10.1371/journal.pone.0020861)
Supplement: Figure S1 — Determination of h SMP30 Transcription start site (TSS). Determination of transcription initiation sites by primer extension analysis. A Lane 1–4, sequencing reactions; lane 5, primer extension product of MCF-7 total RNA, lane 6, labeled DNA marker from Promega. The sequence corresponding to the transcription start site has been marked by a line and is complementary to the sequencing reactions shown in the figure. B. A schematic diagram of human SMP30 promoter showing two TREs, CAAT box and transcription start site. (DOC) [file pone.0020861.s001.doc]

**Supporting Information S1:**

**1. Establishment of hSMP30 transcription start site by Primer Extension analysis**

A 21 nucleotide long primer Xho 1, with the sequence 5’-GGCTGGAAGAATCCTGCAAAG-3’, which is complementary to the +66 to +46 region of SMP30, was used for primer extension analysis. Briefly 5pmole of 5’ end labeled Xho1primer was annealed with 50µg of total RNA at 60ºC for 1h, cooled to room temp and was reverse transcribed at 42ºC for 1hour using primer extension system (Promega, USA) according to manufacturer’s instruction. The same primer was used for the sequencing reactions of cloned SMP30 promoter containing exon 1. Sequencing reactions and primer extension product were separated side by side on a 6% polyacrylamide gel containing 7M urea dried, and autoradiographed.

1. **Determination of hSMP30 Transcription start site**

To determine the transcription initiation site of SMP30, mRNA prepared from MCF-7 cell line was reverse transcribed by ^32^p labelled specific primer and then resolved in a sequencing gel along with the relevant genome sequence. A primer extension product was found 203bp from exon 1 of SMP30 mRNA (Fig.S1**)**


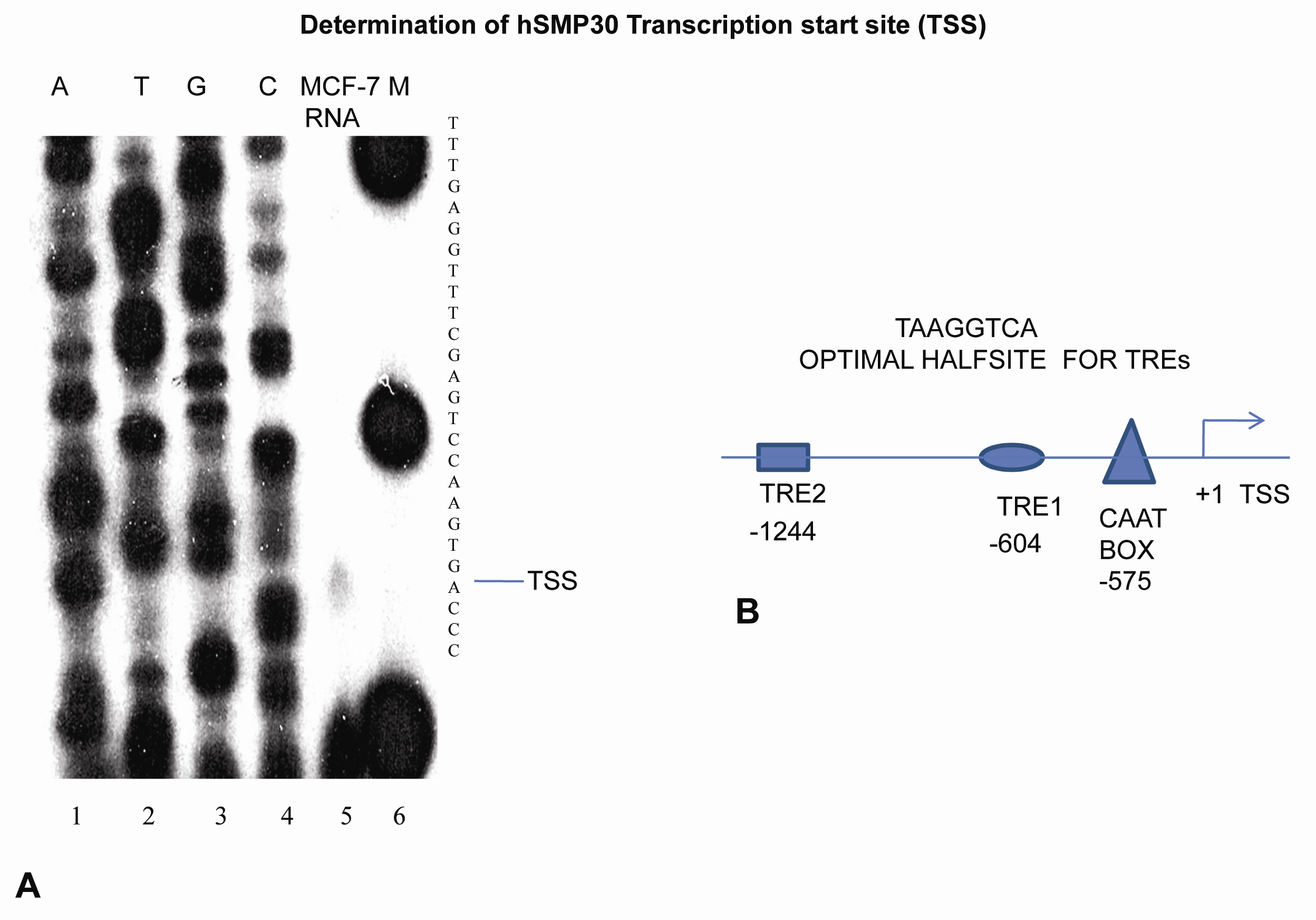


**Figure S1. Determination of hSMP30 Transcription start site (TSS)**

Determination of transcription initiation sites by primer extension analysis.A Lane 1-4, sequencing reactions; lane 5, primer extension product of MCF-7 total RNA, lane 6, labeled DNA marker from Promega. The sequence corresponding to the transcription start site has been marked by a line. A schematic diagram of human SMP30 promoter showing two TREs, CAAT box and transcription start site.
