# Supplementary material for: 3, 3′5 Triiodo L Thyronine Induces Apoptosis in Human Breast Cancer MCF-7cells, Repressing SMP30 Expression through Negative Thyroid Response Elements
Source: PLoS One. 2011 Jun 7;6(6):e20861. doi: 10.1371/journal.pone.0020861 (PMC3110202; doi:10.1371/journal.pone.0020861)
Supplement: Figure S2 — To analyze the effect of T3 on SMP30 Promoter activity in MCF-7 cell in relation to RXRα. Transient transfections of hSMP30 TRE1, TRE2 were carried out using MCF-7 cells. 20 hrs before transfection, cells were plated in DMEM 10%CS media, at a density of 1×10^5^ cells per well in 12 well plates. For transient transfection, 0.5 µg of reporter plasmid DNA, 0.5 µg of TRβ and TRα (TRs), RXRAαexpression vector, 100 ng of pRL-TK control vector and only vector to control cells were co transfected using Fugene HD transfection reagent (from Roche) as per manufacture's instruction. After 24 hrs of transfection, cells were subjected to overnight treatment with 1 µM T3 and vehicle to control cells in 10% CS –DMEM. Then cell lysates were prepared and luciferase activities were measured. Values are the mean of three independent experiments ± SD normalized to Renilla activity. *** P<0.0001difference from vehicle control using ANOVA. (DOC) [file pone.0020861.s002.doc]

**Supporting Information S2:**

**To analyze the effect of T3 on SMP30 Promoter activity in MCF-7 cell in relation to RXRα.** Luciferase activity of both the reporter vectors hSMP30 TRE1 and TRE2were transfected in MCF-7 cells having endogenous TRs did not show any significant difference in presence or absence of T3 where as luciferase activity was induced by over expressing TR alone or along with RXRα in absence of ligand and repressed in presence of ligand as shown in Fig. S2 A, B. However there was no repression found in overexpressing only RXRα by T3 treatment as presented in Fig. S2.


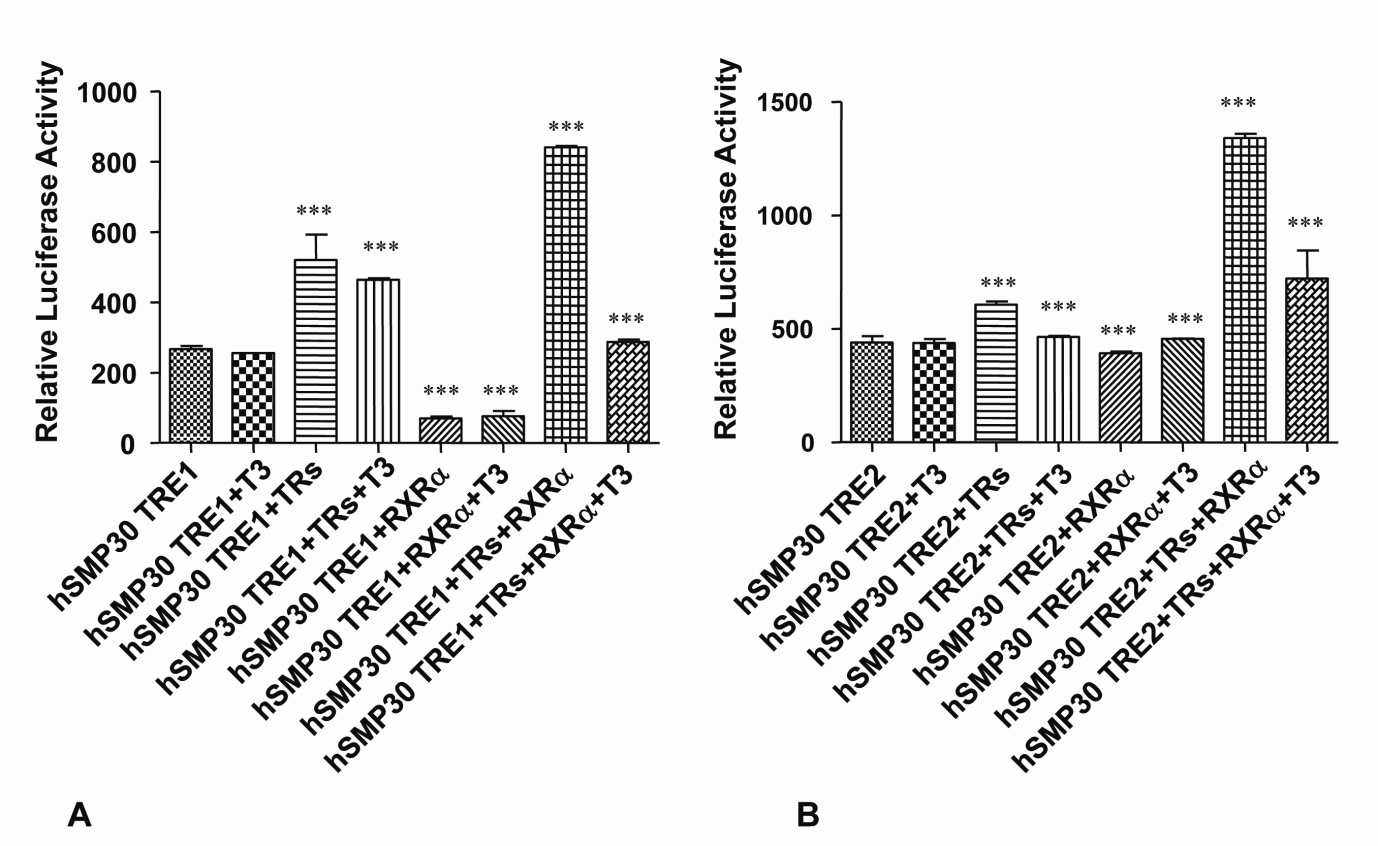
Transient transfections of hSMP30 TRE1, TRE2 were carried out using MCF-7 cells. 20hrs before transfection, cells were plated in DMEM 10%CS media, at a density of 1×10^5^ cells per well in 12 well plates. For transient transfection, 0.5µg of reporter plasmid DNA, 0. 5µg of TRβ and TRα (TRs), RXRα expression vector, 50ng of _p_RL-TK control vector and only vector to control cells were co transfected using Fugene HD transfection reagent (from Roche) as per manufacture’s instruction. After 24hrs of transfection, cells were subjected to overnight treatment with1µM T3 and vehicle to control cells in 10% CS –DMEM. Then cell lysates were prepared and luciferase activities were measured. Values are the mean of three independent experiments ± SD normalized to Renilla activity. *** *P*<0.0001difference from vehicle control using ANOVA.
