# Supplementary material for: 3, 3′5 Triiodo L Thyronine Induces Apoptosis in Human Breast Cancer MCF-7cells, Repressing SMP30 Expression through Negative Thyroid Response Elements
Source: PLoS One. 2011 Jun 7;6(6):e20861. doi: 10.1371/journal.pone.0020861 (PMC3110202; doi:10.1371/journal.pone.0020861)
Supplement: Figure S3 — Luciferase activity of SMP30 TREs in HEK293 cell line. Transient transfections of hSMP30 TRE1, TRE2 were carried out using HEK 293 cells. 20 hrs before transfection, cells were plated in DMEM 10%CS media, at a density of 1×10^5^ cells per well in 12 well plates. For transient transfection, 0.5 µg of reporter plasmid DNA, 0.5 µg of TRβ and TRα (TRs), RXRα expression vector, 100 ng of pRL-TK control vector and only vector to control cells were co transfected using Fugene HD transfection reagent (from Roche) as per manufacture's instruction. After 24 hrs of transfection, cells were subjected to overnight treatment with 1 µM T3 and vehicle to control cells in 10% CS –DMEM. Then cell lysates were prepared and luciferase activities were measured. Values are the mean of three independent experiments ± SD normalized to Renilla activity. ***P<0.0001difference from vehicle control using ANOVA. (DOC) [file pone.0020861.s003.doc]

**Supporting Information S3:**

**The results obtained using other cell line such as HEK 293 cells**

Luciferase activity of SMP30 TRE in HEK293 cellline


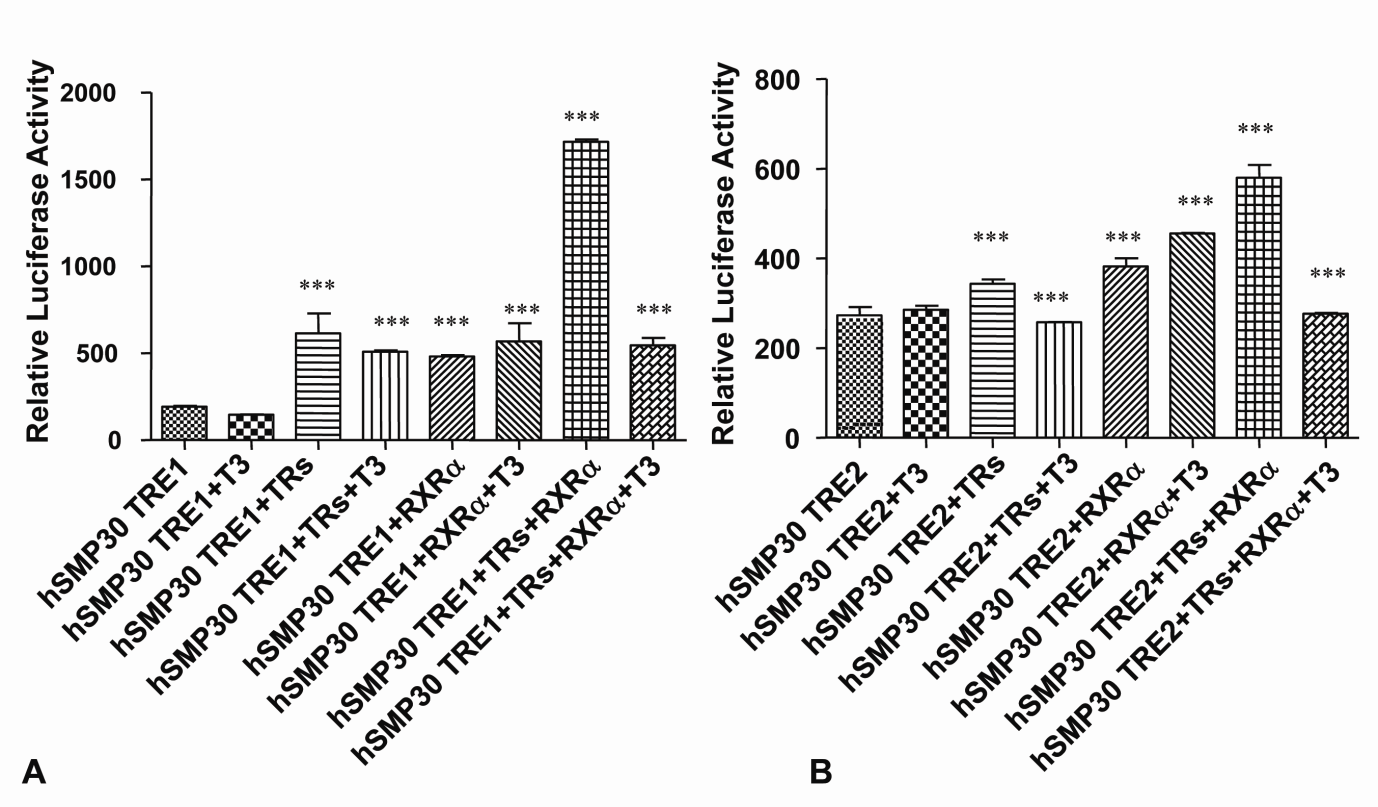


Transient transfections of hSMP30 TRE1, TRE2 were carried out using HEK 293 cells. 20hrs before transfection, cells were plated in DMEM 10%CS media, at a density of 1×10^5^ cells per well in 12 well plates. For transient transfection, 0.5µg of reporter plasmid DNA, 0. 5µg of TRβ and TRα (TRs), RXRα expression vector, 50ng of _p_RL-TK control vector and only vector to control cells were co transfected using Fugene HD transfection reagent (from Roche) as per manufacture’s instruction. After 24hrs of transfection, cells were subjected to overnight treatment with 1µM T3 and vehicle to control cells in 10% CS –DMEM. Then cell lysates were prepared and luciferase activities were measured. Values are the mean of three independent experiments ± SD normalized to Renilla activity. *** *P*<0.0001difference from vehicle control using ANOVA.

We were getting similar pattern of SMP30 TRE promoter activity in presence and absence of thyroid hormone as we found in MCF-7 cellline as shown in Fig. S2.
